# Nest architecture, fungus gardens, queen, males and larvae of the fungus-growing ant *Mycetagroicus inflatus* Brandão & Mayhé-Nunes

**DOI:** 10.1007/s00040-013-0320-8

**Published:** 2013-10-05

**Authors:** A. Jesovnik, J. Sosa-Calvo, C. T. Lopes, H. L. Vasconcelos, T. R. Schultz

**Affiliations:** 1 Department of Entomology, Maryland Center for Systematic Entomology, University of Maryland, 4112 Plant Science Bldg., College Park, MD 20742 USA; 2 Department of Entomology, National Museum of Natural History, Smithsonian Institution, PO Box 37012, MRC 188 CE517, Washington, DC 20013-7012 USA; 3 Instituto de Biologia, Universidade Federal de Uberlândia, C.P. 593, 38400-902 Uberlândia, MG Brazil

**Keywords:** *Mycetagroicus inflatus*, Ants, Attini, Fungus-growing ants, Symbiont fidelity, Nest architecture

## Abstract

All known fungus-growing ants (tribe Attini) are obligately symbiotic with their cultivated fungi. The fungal cultivars of “lower” attine ants are facultative symbionts, capable of living apart from ants, whereas the fungal cultivars of “higher” attine ants, including leaf-cutting genera *Atta* and *Acromyrmex*, are highly specialized, obligate symbionts. Since higher attine ants and fungi are derived from lower attine ants and fungi, understanding the evolutionary transition from lower to higher attine agriculture requires understanding the historical sequence of change in both ants and fungi. The biology of the poorly known ant genus *Mycetagroicus* is of special interest in this regard because it occupies a phylogenetic position intermediate between lower and higher ant agriculture. Here, based on the excavations of four nests in Pará, Brazil, we report the first biological data for the recently described species *Mycetagroicus inflatus*, including the first descriptions of *Mycetagroicus* males and larvae. Like *M. cerradensis*, the only other species in the genus for which nesting biology is known, the garden chambers of *M.* *inflatus* are unusually deep and the garden is most likely relocated vertically in rainy and dry seasons. Due to the proximity of nests to the Araguaia River, it is likely that even the uppermost chambers and nest entrances of *M. inflatus* are submerged during the rainy season. Most remarkably, all three examined colonies of *M. inflatus* cultivate the same fungal species as their congener, *M. cerradensis*, over 1,000 km away, raising the possibility of long-term symbiont fidelity spanning speciation events within the genus.

## Introduction

The ant genus *Mycetagroicus*, described in 2001, is a member of the ant tribe Attini, all of which are obligate agriculturalists and fungivores. In establishing the genus, Brandão and Mayhé-Nunes (2001) included three species: *M. cerradensis*, *M. triangularis*, and *M. urbanus*. In 2008, the same authors described a fourth species, *M. inflatus*, based on two workers collected by R. Feitosa and R. Rosa da Silva in southern Pará, Brazil, in 2005 (Brandão and Mayhé-Nunes, 2008). The descriptions of three of the four *Mycetagroicus* species were based solely on workers. For *M. triangularis*, a single specimen of the gyne was described. Until now, *Mycetagroicus* males and larvae have remained entirely unknown.

*Mycetagroicus* occupies a critical position in the phylogeny of the Attini (Schultz and Brady, 2008). It is the sister group of the so-called higher Attini (a group containing the genera *Trachymyrmex*, *Sericomyrmex*, *Acromyrmex*, and *Atta*) which cultivate a clade of obligately symbiotic fungal species that share a lower attine fungal ancestor, all of which are highly specialized for symbiosis with their ant hosts (Mueller and Gerardo, 2002; Schultz et al., 2005; Mikheyev et al., 2006, 2007; Schultz and Brady, 2008). Based on worker morphology, Brandão and Mayhé-Nunes (2008) suggested that the genus might be a member of the higher Attini, but Solomon et al. (2011), based on a molecular analysis of the cultivar, found that *M. cerradensis* is in fact a member of the lower Attini, i.e., it is associated with a lower attine cultivar. The fungal cultivars of the other three *Mycetagroicus* species have until now remained unknown.

Since the genus is the sister group of the higher attines, study of *Mycetagroicus* species can potentially provide insights regarding the origin of higher attine agriculture, arguably the most ecologically significant transition in the evolutionary history of the Attini. During the past few decades, new natural history information has required periodic, sometimes dramatic revisions of our model of the complex co-evolutionary relationships among fungus-growing ants, their fungal cultivars, and other microbial symbionts (Chapela et al., 1994; Mueller et al., 1998; Currie et al., 1999; Mikheyev et al., 2006, 2007; Mehdiabadi and Schultz, 2010; Mueller et al., 2011a; Mehdiabadi et al., 2012).

Nest architecture has been studied from an evolutionary perspective in a number of different organisms, especially in birds, termites, and social Hymenoptera. In some studies, nest characters were included in a phylogenetic data matrix and analyzed along with other character data to produce a phylogeny (Lanyon, 1986; Wenzel, 1998; Zyskowski and Prum 1999), whereas in other studies, nest characters were mapped onto pre-existing phylogenies to test whether nest traits were correlated with phylogenies obtained from non-nest-related character information (Packer, 1991; Rasmussen and Camargo, 2008). A study of termites in the genus *Apicotermes* Holmgren, 1912 included a key to the species based solely on nest morphology (Schmidt, 1955), concluding that termite nests contain more phylogenetic information than the termites themselves. In ants, studies of underground nests (Tschinkel, 2003) and of nest entrance morphology (Schultz et al., 2002) have revealed patterns in structure and morphology that are species-specific. At deeper phylogenetic levels, however, due to convergence and parallelism nest architecture characters are often homoplastic, arising multiple times on the ant tree of life (Tschinkel, 2011). Nest architecture in fungus-growing ants is important not only for the ants but also for their fungal cultivars, which require particular environmental conditions (Bollazzi and Roces 2002; Bollazzi et al. 2008). Nest architecture, therefore, is a significant aspect of attine ant biology, and the importance of documenting attine nest life histories has been recognized by multiple researchers (Weber, 1972; Solomon et al., 2004, 2011; Moreira et al., 2004; Fernández-Marín et al. 2005; Diehl-Fleig and Diehl, 2007; Klingenberg et al., 2007; Rabeling et al., 2007; Mehdiabadi and Schultz, 2010; Ramos-Lacau et al., 2012).

In addition to describing nest architecture, we identify the fungal cultivar of *Mycetagroicus inflatus*. We describe the previously unknown queen, provide the first description of a male for the genus, and compare both to reproductive forms of other, closely related attine genera. We also describe for the first time the larva of a *Mycetagroicus* species.

## Materials and methods

Field work was conducted from 5 to 10 October 2012 in eastern Pará State, Brazil, at the end of the dry season. During this time period the first rains of the rainy season occurred. All nests were found at two localities on the west coast of the Araguaia River, a southern component of the Amazon River Basin, across from the town of Araguacema, Tocantins, in an area characterized as “dense alluvial forest” (Silva, 2007). At “Locality 1” (S 08.75387°, W 49.54544°, elevation 164 m), nest entrances occurred approximately 15 m from the river’s edge in an area about 7 m above water level, which was at its seasonal low point; at “Locality 2” (S 08.80157°, W 49.57368°, elevation 176 m), they occurred approximately 5 m away from the river’s edge and about 5 m above the water level. At both localities the beach area is sandy, with occasional low bushes, and banks are covered with gallery forest. Based on information from local inhabitants, both Locality 1 and Locality 2 are entirely submerged during the rainy season (December–March).

In both localities, nests were located in the morning by following foraging workers returning with bait (Cream of Rice cereal) to their nest entrances. Nests were excavated using Gerber folding shovels by first digging a trench approximately 1.5 m away from the nest entrance (Rabeling et al., 2007; Solomon et al., 2011). The trench was extended by carefully shaving away soil with a shovel or a knife in the direction of the nest entrance. As the trench grew closer to the nest entrance, digging became increasingly careful in order to avoid missing or destroying chambers and tunnels. Since the depths at which *M. inflatus* constructs its deepest chambers were initially unknown, after locating a chamber excavation continued downward 0.5–1 m in order to locate possible additional chambers. If a chamber contained a queen, it was assumed that it was the bottom-most chamber and excavation ceased. When a chamber was located, part of the chamber wall near the excavator was removed and the fungus garden, when present, was transferred, using a flame-sterilized forceps, spoon, and/or knife, to a plastic nest box with a plaster bottom pre-saturated with water. Ants were collected with sterilized soft forceps and with an aspirator. Chamber dimensions were recorded, including: height (maximum length along vertical axis), width (maximum length along horizontal axis parallel to the excavation plane), depth (maximum length along horizontal axis perpendicular to the excavation plane), and distance from the surface to the chamber roof (summarized in Table 1). Nests were maintained in live laboratory culture and were still alive in the lab at the time of this writing (July 2013). Several workers were preserved immediately in 95 % ethanol for later DNA extraction. A few days following collection, small portions of fungus gardens were collected from the live lab nests and likewise preserved in 95 % ethanol. The time interval between field collections of the nests and preservation of garden samples allowed the ants to reassemble the gardens, which are usually broken apart during collection, and to remove particles of soil, which are usually introduced during nest excavation. Estimated number of individuals for each colony is based on the count of the workers in the laboratory nests; it may be an underestimate because laboratory colonies appear to have fungus gardens that are smaller than those initially collected in the field.

**Table 1:** Summary of nest data

Nest ID		Chamber 1	Chamber 2	Chamber 3	Chamber 4
JSC121006-02 Locality 1	Depth	80 cm	94 cm	103 cm	\
	Dimensions	6.5 × 4 × 5 cm	5 × 4 × 7 cm	7.5 × 6 × 7 cm	
	Contents	fn, w, al	fn, w, al	fn, w, al, q	
TRS121006-07 Locality 1	Depth	22 cm	68 cm	83 cm	96 cm
	Dimensions	4 cm	2 cm	7 cm	7 × 8 cm
	Contents	Loose sand	w, m	w, al	fn, w, g, m, 2q
TRS121009-01 Locality 2	Depth	87 cm	100 cm	230 cm	\
	Dimensions	8 × 4 × 10 cm	8 × 5 × 9 cm	3 × 3 × 3.5 cm	
	Contents	w	fn, 1q?	1q?	
TRS121009-02 Locality 2	Depth	75 cm	310 cm	\	\
	Dimensions	9 × 5 × 12 cm	7 × 4 × 7 cm		
	Contents	fn, w	fn, w, m		

Dimensions: width × height× depth

*fn* fungus garden, *w* workers, *q* queen, *m* males, *g* alate females, *al* alates (sex not recorded); for nest TRS121009-01,* 1q?* indicates that we did not record in which chamber the queen was found

In the lab, a few strands of hyphae from 3 different nests (collection codes JSC121006-02, TRS121006-07 and TRS121007-01) were separated from the field-preserved garden material with flame-sterilized forceps under a stereomicroscope and DNA was extracted from them using the Chelex protocol of Sen et al. (2010). The extracted DNA was amplified and sequenced for the nuclear ITS region following the methods of Mueller et al. (1998). This DNA sequence was incorporated into a large alignment (>440 sequences) of fungal cultivars and free-living Leucocoprineae and aligned using MAFFT (Katoh and Standley, 2013) using parameter values E-INS-i, 200PAM/k = 2, and Gap open penalty = 1.2. The sequences are deposited in GenBank under accession numbers KF562343, KF562444, and KF562345.

In the laboratory, ant specimens were examined and measured using SZH Olympus and MZ16 Leica stereomicroscopes. Specimens were photographed using a JVC KY-F70B video camera with M420 Leica stereomicroscope and Automontage Pro version 5.03.0018 software. Larval morphology was examined with scanning electron microscopy (SEM). Five specimens were studied, including three last-instar worker larvae and two worker prepupae (i.e., post-feeding last instars), all taken from nest collection TRS121006-07. Morphological terminology and measurement indices follow Snodgrass (1910), Tulloch (1935), Gauld and Bolton (1988), Hölldobler and Wilson (1990), Bolton (1994), Schultz and Meier (1995), Mackay et al. (2004), Klingenberg and Brandão (2009), and Sosa-Calvo and Schultz (2010). When terminologies disagree, synonymous terms are indicated in parentheses. Morphological measurements, index abbreviations, and definitions are as follows: head width (HW): in frontal view, maximum width of the head just above eyes, excluding eyes; head width in males (HWm): in frontal view, maximum width of the head including eyes; interocular distance (IOD): in the male, maximum width of the head in frontal view measured at the midpoint of the internal margin of eyes; head length (HL): in frontal view, maximum length of the head from posteriormost margin of the head to median point of anteriormost margin of clypeal apron; scape length (SL): maximum length of scape excluding the basal condyle; interfrontal width (IFW): maximum distance between lateral margins of frontal lobes; mandible length (ML): in frontal view, straight-line length from midpoint of anterior margin of clypeal apron to tip of mandibles when fully closed; eye length (EL): in profile, maximum diameter of eye; Weber length (WL): in lateral view, length of mesosoma from anteriormost point of pronotum, to posteriormost ventral angle of propodeum; hind femur length (HfL): length of hind femur in lateral view; petiole length (PL): in lateral view, maximum length of petiole; postpetiole length (PPL): in lateral view, maximum length of postpetiole; gaster length (GL): in lateral view, distance from anteriormost point of tergo-sternal gaster suture to the posterior tip; cephalic index (CI): for females (HW/HL) × 100, for males (IOD/HL) × 100; frontal lobes index (FLI): for females (IFW/HW) × 100, for males (IFW/IOD) × 100; scape index (SI): for females (SL/HW) × 100, for males (SL/IOD) × 100.

Deposition of material: The specimens examined are deposited in USNM, National Museum of Natural History, Washington, DC, USA; MZSP, Museu de Zoologia da Universidade de São Paulo, São Paulo, SP, Brazil; MBC–UFU, Museu de Biodiversidade do Cerrado, Universidade Federal de Uberlândia, Uberlândia, Minas Gerais, Brazil; and MCZ, Museum of Comparative Zoology, Harvard University, Cambridge, MA, USA.

## Results

### Nest architecture

**Fig. 1:**
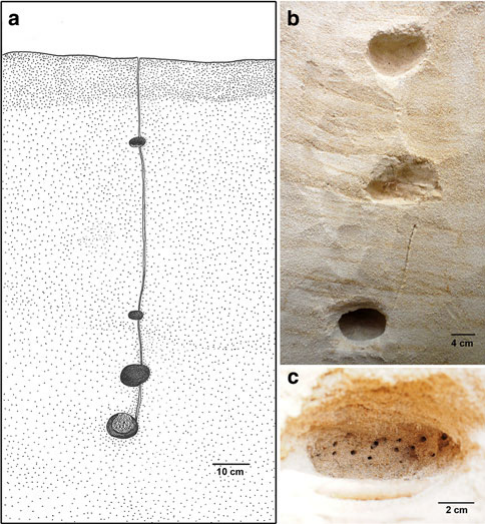
*Mycetagroicus inflatus* nest: **a** Illustration of nest TRS121006-07. **b** Photo of excavated nest JSC121006-02. **c** Photo of chamber 1 of nest TRS121009-02 showing the many holes punctuating the chamber wall

Four nests of *Mycetagroicus inflatus* were excavated. All nests had a single, simple, very inconspicuous entrance hole approximately 2–3 mm in diameter. In no case was the nest entrance surrounded by a mound of excavated soil particles, differing in this regard from entrances of the congeneric *M. cerradensis* (Solomon et al., 2011). Nests contained 2–4 chambers, which varied from 2–8 cm in width. The deepest chamber encountered was 310 cm deep and contained garden and ants, but no queen. The shallowest chamber encountered was 22 cm deep and contained no fungus or ants, only loose sand. The shallowest chamber that contained ants was 68 cm deep, and the shallowest chamber that contained fungus garden was 75 cm deep. Tunnels connecting chambers were observed in some cases and were very straight and perpendicular to the surface (Fig. 1). The walls of two nest chambers, from nests TRS121009-01 and -02, were punctuated with multiple holes (Fig. 1c). Colony sizes ranged from approximately 30–100 workers.

One chamber of a fifth nest was encountered accidentally (TRS121007-01) while digging, but no data on the position of the nest entrance or other chambers were available for that nest, so it is not treated as a separately excavated nest in Table 1. This isolated chamber was encountered at 80 cm depth, was 6 cm wide, and contained a fungus garden, queen, workers, and brood.

### Fungal symbiont

ITS sequences indicate that the fungal cultivars collected from three nests of *M. inflatus* (JSC121006-02, TRS121006-07, and TRS121007-01) all belong to subclade F of the so-called “Clade 2” of lower attine (G3) leucocoprineaceous cultivars (Mehdiabadi et al., 2012; Mueller et al., 1998). Subclade F is arguably a single fungal species that is also associated with *Cyphomyrmex faunulus*, *Mycocepurus smithii*, *Myrmicocrypta* cf. *buenzlii*, and other attine species from Ecuador, Trinidad, Guyana, and Brazil (Mehdiabadi et al., 2012; unpubl. data). Remarkably, the fungal species cultivated by *M. inflatus* in Pará is also grown by its congener *Mycetagroicus cerradensis* (Solomon et al., 2011, accession number in GenBank HM245775) over 1,000 km to the south in Minas Gerais.

### Arthropod symbiont

While following a *M. inflatus* forager carrying Cream of Rice bait back to its nest, a fly, later identified as belonging to the genus *Pholeomyia* Bilimek (Milichiidae: Diptera), was observed following the worker by walking a short distance behind it (JSC, pers. obs.). Sabrosky (1959) reported similar observations by W.L. Brown and E.O. Wilson of a *Pholeomyia* fly following a worker of the fungus-growing ant *Trachymyrmex septentrionalis* McCook in Florida and suggested a possible association of certain *Pholeomyia* species with fungus-growing ants. Later, Waller (1980) reported finding *Pholeomyia texensis* Sabrosky entering the nests of *Atta texana* by riding on the cut leaves, where the fly larvae presumably feed on nest refuse. Larvae of some species of milichiids have close associations with Hymenoptera (Sabrosky, 1959; Krombein, 1967; Moser and Neff 1971; Melo, 1996; Wild and Brake, 2009; Swann, 2010).

### Systematic treatment

*Mycetagroicus inflatus*, Brandão & Mayhé-Nunes 2008

*Gyne* (*Fig*. 2)

**Fig. 2:**
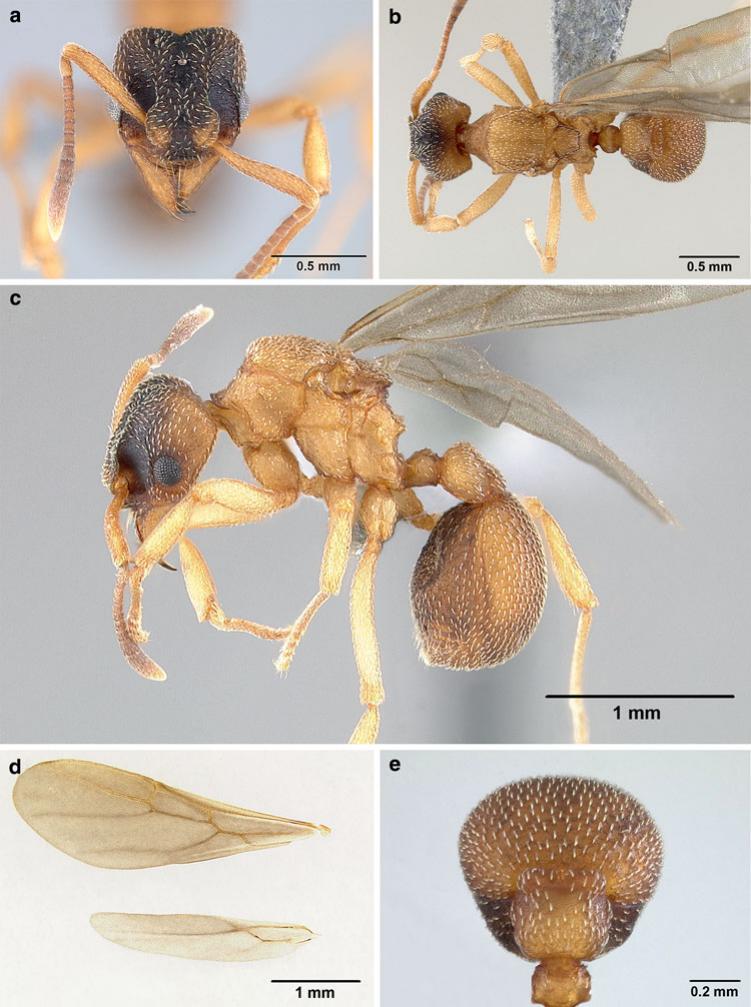
*Mycetagroicus inflatus* gyne: **a** Full-face view, **b** dorsal view, **c** lateral profile, **d** fore- and hindwing, **e** dorsal view of postpetiole

Measurements in mm (range):

HW 0.60–0.72, HL 0.72–0.76, ScL 0.62–0.66, IFW 0.38–0.39, ML 0.20–0.26, EL 0.15–0.17, WL 1.0–1.1, HfL 0.79–0.87, PL 0.18–0.23, PPL 0.32–0.37, GL 0.89–1.14, CI 84–94, FLI 55–62, SI 91–102 (*n* = 7).

Posterolateral corners of head, mesosoma, petiole, and postpetiole light ferruginous brown; gaster dark ferruginous brown. Entire dorsum of head in full-face view fuscous, darker than gaster. Mandibles, antennae, and anterior borders of frontal lobes distinctly lighter than the rest of the dorsum of the head. Integument finely reticulate-punctuate, opaque. Erect hairs absent, most of the body covered with short, white, appressed hairs.

*Head*. In full-face view slightly longer than broad (CI 84–94), posterior corners of cephalic margin rounded, slightly notched medially. Mandibles triangular, dorsally striate, masticatory (inner) margin of mandible with 5 teeth, the distal-most tooth noticeably prolonged and larger than the rest. Outer margin of mandible straight. Margin of anterior clypeal apron convex, with a very broad and shallow but distinct median notch. Median clypeal seta (~0.15 mm) arising from border of clypeal apron and clypeal anterior margin, flanked on either side by a pair of additional, long setae as well as 2–3 shorter setae. Distinct frontoclypeal teeth present (“triangular lateral tooth” of Brandão and Mayhé-Nunes, 2008), best seen in lateral view. Frontal lobes evenly rounded, separated by a fingerlike extension of the clypeus extending posterad to the level of the eyes. Frontal carinae short, diverging toward corners of the head, not extending beyond median ocellus. Preocular carina distinct, extending posterad from mandibular insertion, then curving medially above eye level. Area laterad of frontal lobes, the ill-defined antennal scrobe, devoid of hairs. Eyes convex, 13 ommatidia across largest diameter of dorso-ventral axis and 10 ommatidia across smallest diameter. Three ocelli present, all similar in size. Antennal scape extending only slightly (~0.02 mm) past posterior border of head. Scape noticeably curved ventrad in basal one third, best seen in posterior view with scape positioned at 90° from median axis. Antenna 11-segmented, lacking distinctive antennal club. First funicular segment and segments 8–10 longer than broad, segments in between almost subquadrate. Palp formula 4, 2.

*Mesosoma*. Lateral pronotal tubercle present, dentiform. Anterior pronotal tubercle absent (“paired median pronotal teeth” of Brandão and Mayhé-Nunes, 2008). Antero-inferior corner of pronotum almost forming a right angle, best seen in lateral view. Anapleural suture present (“median episternal groove” of Snodgrass, 1910). Scutum lacking notauli or other sutures, shallowly impressed longitudinally. Parapsidal furrow absent. Axillae (“anterior division of scutellum” sensu Snodgrass, 1910; “prescutellum” sensu Tulloch, 1935; “paraptera” sensu Brandão and Mayhé-Nunes, 2008) relatively large, laterally rounded and medially constricted. Scutellum mostly flat in profile view, weakly concave and narrowing posteriorly in dorsal view, posterior border medially emarginate, not forming scutellar processes. Propodeal teeth short, blunt, and directed posterolaterally. Propodeal carinae absent. Propodeal spiracle directed posterad, mounted on a tumulus, best seen in dorsal view. Propodeal lobe lamellate, connected by a short, longitudinal carina, along bulla of metathoracic gland, to the dorsum of the propodeal spiracle, best seen in dorsolateral view.

*Wings*. Transparent, covered with minute pilosity, veins brown. Forewing (length: 3.62 mm) with 5 closed cells (terminology follows Goulet and Huber, 1993): costal (C), radial (R), cubital (Cu), first radial 1 (1R1), and first radial 2 (2R1). Pterostigma not visible. Hindwing (length: 2.57 mm) with reduced venation, just one closed cell, and 6 hamuli.

*Metasoma*. Petiole compact, relatively small, petiolar peduncle vestigial. Subpetiolar process present anteriorly, pointing forward, sometimes not clearly visible because concealed by propodeal lobes. From ventral view clearly visible as a spine. Petiole dorsum with two small denticles near its posterior border. Postpetiole large, >1.5× the width of the petiole, slightly broader than long in dorsal view. Posterior border of postpetiole wider than the rest of petiole, bearing protruding transverse plate that is rounded laterally and slightly emarginated medially (Fig. 2e). In dorsal view, gaster elliptical, narrowing posteriorly, lacking ridges or noticeable sculpture, but having finely punctuated microsculpture. Rows of long, suberect hairs present on posterior borders of gastral sternites 2–4 (A5–A7). Gastral tergite and sternite I subequal in length, the posteriormost tip of the gaster formed by segment A7 in contrast to the (presumably derived) condition in some Attini, in which gastral tergite I longer than sternite I so that posteriorly it overlaps the remaining segments, which are shifted anterad.

*Differential diagnosis.* The gyne of *M. inflatus* is, as in many ant species, quite similar to the worker except for modifications typical for the caste such as the presence of ocelli, the morphology of the mesosoma associated with wings, and the slightly larger size. In addition, in the worker the eyes are smaller, the frontoclypeal teeth are less pronounced, the frontal lobes are narrower, median anterior tubercles are present on the pronotum, and the petiole is shorter. The gyne of *M. inflatus* can be distinguished from the gynes of other closely related attine genera by characters that are synapomorphic for the genera. *Sericomyrmex* gynes, for instance, are larger in size and covered with dense pilosity, have cordate heads, have gasters that are laterally straight instead of rounded, and have frontal lobes that are more developed than those in *Mycetagroicus*. Gynes of *Trachymyrmex* species are in most cases larger in size and have integuments that are moderately to strongly tuberculate, frequently appearing spiny, most noticeably on the posterior lobes of head and on the gaster, which in *M. inflatus* are smooth. *Cyphomyrmex* gynes can be similar in size to gynes of *M. inflatus,* but are immediately distinguished by the strongly expanded frontal lobes, often covering a large portion of the head, and in the *strigatus* and *wheeleri* groups accompanied by deep antennal scrobes. The gyne of the congeneric *M. triangularis* can be distinguished from that of *M. inflatus* by its slightly larger size, the presence of a median longitudinal ridge posterior to the frontal triangle, the presence of a distinct parapsidal furrow, the presence of scutellar processes, and the rugulose microsculpture on the dorsum of first gastral segment (A4), which also bears two pronounced ridges laterally.

*Male* (Fig. 3)

**Fig. 3:**
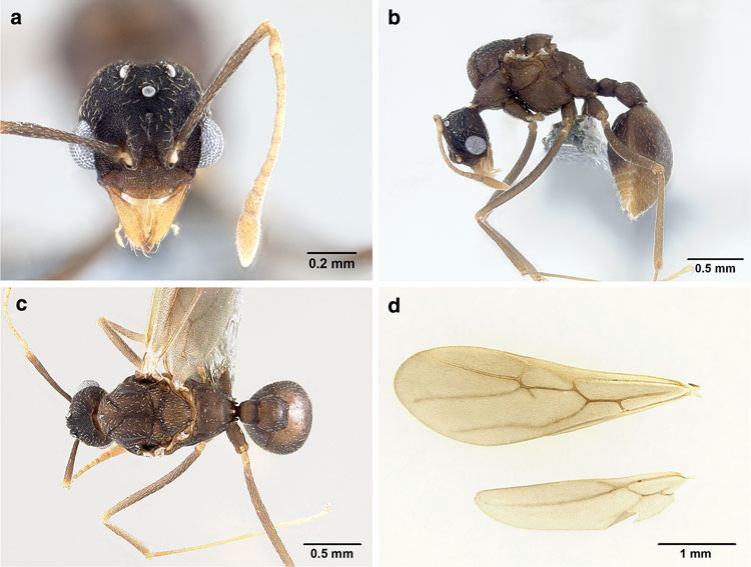
Male of *Mycetagroicus inflatus*: **a** full-face view, **b** lateral profile, **c** dorsal view, **d** fore- and hindwings

Measurements in mm (range):

HWm 0.53–0.61, IOD 0.37–0.40, HL 0.45–0.56, ScL 0.44–0.62, IFW 0.15 0.19, ML 0.14–0.21, EL 0.13–0.19, WL 0.90–1.16, HfL 1.05–1.35, PL 0.14–0.20, PPL 0.21–0.29, GL 0.80–1.07, CI 77–81, FLI 41–44, SI 120–142 (*n* = 7).

Body color dark brown, integument reticulate-punctuate on head and mesosoma, gaster opaque with reticulate microsculpture. Erect hairs absent, short appressed white hairs covering head, dorsal side of mesosoma, gaster, and legs; only a few hairs present on lateral side of mesosoma.

*Head*. In full-face view longer than broad, posterior border smoothly rounded. Mandibles triangular, dorsally striate, basal angle rounded, masticatory margin with three teeth, all arising in the distal half, an unusual arrangement shared with males of *Trachymyrmex urichi* and perhaps other *Trachymyrmex* species. Clypeus evenly reticulate, without frontoclypeal carinae or teeth as seen in worker and queen. Anterior margin of clypeal apron convex, with shallow median notch. Median clypeal seta (~0.12 mm) arising at the point where posterior margin of clypeal apron and anterior clypeal margin meet flanked by one much shorter seta on each side. Frontal lobes small, short, not completely covering the antennal condyle, broadly separated by a fingerlike extension of the clypeus extending posterad. Frontal carinae short, extending to the level of the posterior border of the eyes. Preocular carina distinct, curving posteriorly toward the midline. Eyes convex, large, 16 ommatidia across largest diameter of dorso-ventral axis, 15 ommatidia across the smallest diameter of the eye. Three similarly sized ocelli present. Antennal scape straight, long, extending well beyond the occiput. Antenna 12-segmented, a departure from the plesiomorphic number of 13 for ant males (including attine males), the first funicular segment (pedicel) as long as second funicular segment, or slightly longer. First funicular segment longer than broad and thicker than the second funicular segment, but not thicker than the most distal segments. Palp formula 4, 2.

*Mesosoma*. Lateral pronotal tubercles absent. Antero-inferior corner of pronotum rounded. Anapleural suture present, dividing mesopleuron into katepisternum and anepisternum. A wide, transversely costate groove present on dorsoposterior border of anepisternum below insertion of wing. In some males the anapleural suture can also be transversely costate. Scutum with shallow, complete, V-shaped notauli, dividing the scutum into an anteromedian area (“prescutum”) and two lateral areas. Median mesoscutal sulcus present, fading posteriorly. Parapsidal lines present. Axillae relatively large, rounded laterally, constricted medially. Scutellum slightly inflated, narrowing posteriorly, posterior border with two short, blunt denticles. Propodeal teeth reduced to short, blunt denticles.

*Wings*. Forewing (length: 3.81 mm), hindwing (length: 2.7 mm). Venation and appearance same as in female, except in male spur of cross vein 1 m-cu is visible protruding from the bottom of 1R1 cell and 7 hamuli instead of 6 on hind wing.

*Metasoma*. Petiole compact, node in dorsal view rounded, slightly broader than long. Petiolar sternite narrowing to a forward directed, sharp keel. Postpetiole in dorsal view rounded, slightly broader than long, posteriorly emarginate. Integument of tergum 1 of gaster (i.e., A4) more shining than the other body parts, but still subopaque due to the finely reticulate microsculpture; bearing sparse, white, appressed hairs. In dorsal view, gaster elliptical, narrowing posteriorly, without any carinae or other macrosculpture. Gastral tergite and sternite I subequal in length and gastral tergites 2–5 (i.e., A5–A8) visible dorsally or posterodorsally. Rows of long, suberect hairs present on posterior borders of gastral sternites 2–5 (A5–A8).

*Differential diagnosis.* Males of *M. inflatus* have 12-segmented antennae, a deviation from the usual 13-segmented antennae present in the males of most attine species, although the 12-segmented condition has arisen independently elsewhere in the tribe in the genus *Sericomyrmex*, in some *Cyphomyrmex* species (e.g., *C. faunulus* and *C. auritus*), in *Trachymyrmex opulentus*, and in a number of social parasites in the genus *Acromyrmex* (Gallardo, 1916). Reductions in male antennal segment number are even more dramatic in males of the socially parasitic attine species *Mycocepurus castrator* (Rabeling and Bacci, 2010) and *Pseudoatta argentina* (Gallardo, 1916; Schultz et al., 1998), which have 11-segmented antennae. The male of *M. inflatus* can be distinguished from males of closely related genera by the following combination of characters: presence of 12 antennal segments, reticulate-punctuate integument (even on the clypeus), dark brown color, transversely costate groove on the mesopleuron, and propodeal teeth very reduced. Males of *Sericomyrmex* can be distinguished from males of *M. inflatus* by the presence of dense appressed pilosity covering the whole body and longer, thicker, decumbent to erect hairs on the head, mesosoma, and dorsum of the gaster. Males of *Trachymyrmex* usually also have denser pilosity, are sometimes covered with spiny tubercules on the head and gaster, and usually have sharp propodeal spines and humeral tubercles. Males of the closely related species *Cyphomyrmex costatus* and *C. wheeleri* have 13 antennal segments, strongly developed frontal lobes, antennal scrobes that reach the posterior border of the head, and sharp propodeal spines.

*Larva* (Fig. 4)

**Fig. 4:**
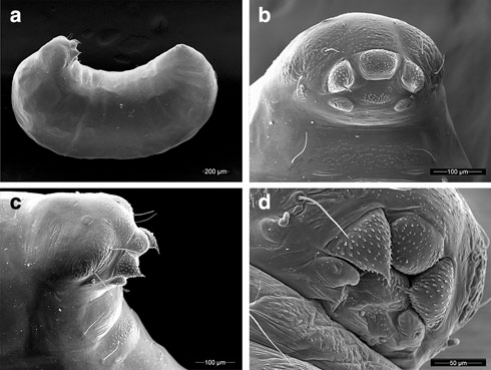
Larvae and prepupae of *Mycetagroicus inflatus*: **a** lateral profile (prepupae), **b** head (prepupae), **c** head, lateral view (prepupae), **d** mouthparts (last-instar larvae)

Specimens examined: three last-instar worker larvae and two worker prepupae (i.e., post-feeding last instars), all from nest collection TRS121006-07. Profile “attoid” sensu Wheeler and Wheeler 1976, i.e., with a moderately curved, ventrally shortened profile. As in all other Attini, thoracic-abdominal articulation absent, thoracic intersegmental constrictions superficial, deep lateral depressions associated with abdominal spiracles absent, and leg vestiges present as open slits. As in most other Attini, dorsal and lateral body surfaces devoid of setae. Differing from other Attini in the remarkably small number of head and ventral setae. Head devoid of setae except for two setae on each gena, a state previously unknown in attine larvae; ventral thoracic segments 1–3 each with a single pair of setae laterally; and two short setae on abdominal segment 10 ventral to the anus. Ventral thoracic segments 1 and 2 medially bearing multidentate spinules, thoracic segment 2 additionally with a low ventromedian boss, in combination with the genal and thoracic setae clearly functioning as a food anchor. As in most Neoattini, genal lobes present. Labrum monolobate, narrow, bulging, a synapomorphy for the Attini; anterior labral setae reduced to papillae. Mandibles typically attine: short, fleshy, subconical. A distinct, undivided apical mandibular tooth and no subapical teeth; spinules sparsely but evenly distributed on all mandibular surfaces. Mandibular gnathobases absent. Basal portions of maxillae fused with head capsule. As in all other Neoattini, maxillary palp widely removed laterad from galea. Galea reduced, present as two sensilla surmounting a low welt; a shallow pit distal and immediately adjacent to the galea. Maxillary palp digitiform, maxillary accessory palpal sensillum absent. One seta on each maxilla between the galea and palp, a second seta present laterad of the palp. As in most attines, labium feebly protruding and lateral sericteral protuberances absent; labial palps papilliform. Labial spinules present basad and absent distad of the sericteries. Hypopharyngeal spinules largely unidentate and moderately to sparsely distributed.

## Discussion

The genus *Mycetagroicus* is the sister group of the higher Attini (*Sericomyrmex*, *Trachymyrmex*, *Acromyrmex*, and *Atta*) (Schultz and Brady, 2008). Thus, understanding the biology of *Mycetagroicus* is our best hope for reconstructing the attributes of the most recent common ancestor that it shares with the higher Attini, especially when considered in combination with the biology of the most closely related outgroup [currently thought to be the *Cyphomyrmex wheeleri* clade (Schultz and Brady, 2008)]. Reconstructing the common ancestor of *Mycetagroicus* + the higher Attini is important for reconstructing the evolutionary transitions that occurred on the branch subtending the higher Attini, arguably the most ecologically significant period of evolutionary change in all of the Attini. These transitions coincide with the origin of the obligate higher attine fungal symbiont, derived from a lower attine fungal species (Schultz et al., 2005; Mikheyev et al., 2006, 2007; Mueller et al., 2010; Mueller et al., 2011a). This study adds to the scant biological information so far accumulated for the four known *Mycetagroicus* species. It describes the gyne of *M. inflatus*, increasing to two the numbers of species for which gynes are known, the other being *M. triangularis* (Brandão and Mayhé-Nunes, 2001). It describes for the first time *Mycetagroicus* males and larvae. The nest architecture of *M. inflatus* is also described, increasing to two the number of species for which nest architecture is known, the other being *M. cerradensis* (Solomon et al., 2011).

All four excavated nests of *Mycetagroicus inflatus* had similar characteristics. The walls of one chamber in each of the two nests from Locality 2 (TRS121009-01: chamber depth not recorded; TRS121009-02: chamber 75 cm deep) were punctured by numerous holes of unknown purpose (Fig. 1c). It is possible that such punctures were present in additional chambers but were overlooked. It is highly unlikely that all of the holes were the openings of tunnels because no ants were observed entering or exiting and, if they were tunnel openings, they would connect with dozens of horizontal tunnels whereas no such tunnels were encountered; all of the tunnels observed during excavation were vertically arranged. One possibility is that the holes are somehow associated with flooding, perhaps serving as shelters for the ants during the months that the nests are submerged or serving in some way to capture and hold pockets of air. This conjecture gains support from the observation that similarly punctured chamber walls, as described above, were observed in the deepest chambers of colonies of the sympatric but very distantly related species *Kalathomyrmex emeryi*, colonies of which are certainly flooded during the rainy season. In addition to being punctured by numerous holes, unlike those of *M. inflatus* the chamber walls of *K. emeryi* were lined with a layer of brown clay.

The nest architecture of *Mycetagroicus cerradensis,* the only other congeneric species for which nesting biology is known, shares some characteristics with that of *M. inflatus,* including: a single nest entrance, chambers that are more or less vertically arranged below the nest entrance, and an extremely deep lowermost garden chamber (Solomon et al., 2011). Solomon et al. (2011) suggested that *M. cerradensis* moves its fungus garden seasonally, using deeper chambers in the dry season and shallower chambers in the wet season. This hypothesis applies equally to *M. inflatus* because, in both species, nests were excavated in the dry season and the uppermost nest chambers were found to be empty. Seasonal repositioning of fungus gardens has been recorded for some other fungus-growing ants, including two species of the genus *Mycocepurus* (Rabeling et al., 2007) and in the North American species *Atta texana* and *Acromyrmex* *versicolor* (Moser, 1962, 2006; Mueller et al., 2011a, b).

Perhaps the most potentially important result of this study is the identification of the fungal cultivars grown by *M. inflatus* at the study sites on the Araguaia River. The gardens from all three collected nests belong to the same fungal species (Clade 2, subclade F; Mehdiabadi et al., 2012), suggesting that *M. inflatus* may depart from most lower attine species in possessing a high degree of symbiont fidelity. Even more surprising, *M. inflatus* cultivates the same fungal species as its congener *M. cerradensis* in Minas Gerais, Brazil, over 1000 km to the south (Solomon et al., 2011). Field research during the past two decades has established that attine cultivars are frequently transmitted horizontally between colonies, including colonies of different ant species, and that free-living fungi are frequently imported into the lower attine symbiosis as well (Mueller et al., 1998; Vo et al., 2009; Green et al., 2002; Mueller et al., 2001; Mikheyev et al., 2008). These observations have led to the current paradigm of “diffuse” attine ant/fungus coevolution (Mikheyev et al., 2006; Mikheyev et al., 2007; Mikheyev et al., 2010) and a general expectation of weak symbiont fidelity and frequent reassociations of ants and fungi in attine agriculture. One exception to this rule is the *Cyphomyrmex wheeleri* group, in which it has recently been shown that particular ant and fungal species have been associated for many millions of years (Mehdiabadi et al., 2012). *Mycetagroicus* may represent a second such exception for reasons mentioned above. Interestingly, the genus *Mycetagroicus* is the sister group to the higher Attini and the *C. wheeleri* group is thought to be the sister group to the clade containing *Mycetagroicus* and the higher Attini, suggesting that an increase in symbiont fidelity may have preceded the origin of the higher Attini. Although based on only four samples, this pattern is consistent with a scenario of strong symbiont fidelity that spans speciation events. Obviously, additional samples are necessary for judging whether this pattern of symbiont fidelity continues to hold or whether the observed association with the same fungal species within and across species of *Mycetagroicus* is due to a rare coincidence.

Symbiont choice is favored when potential symbionts vary in their abilities to enhance partner fitness. *Mycetagroicus* species live in habitats that may be at least seasonally inhospitable to free-living leucocoprineaceous fungi. Three of the four species in the genus *Mycetagroicus* are found in the Cerrado; the fourth, *M. inflatus*, is so far known from seasonally flooded riverbanks in a region characterized as “dense alluvial forest” (Silva, 2007). In such habitats, it may be the case that some fungal symbionts are better adapted than others, providing conditions that favor symbiont choice and long-term, close associations of particular ant and fungal species. Arguing against this, however, is the observation that most *Cyphomyrmex wheeleri* group ant species live in wet forests where feral leucocoprineaceous cultivars are known to commonly occur (Mueller et al., 1998) and in which nests of different ant species, each containing its particular fungal symbiont species, often co-occur within centimeters of one another (Schultz et al., 2002). Clearly, more data are required for understanding the mechanisms that produced the genetic and morphological modifications encountered today in the spectacularly successful higher attine fungi and ants.

## References

[CR1] Bollazzi M., Kronenbitter J. and Roces F. 2008. Soil temperature, digging behaviour, and the adaptive value of nest depth in South American species of *Acromyrmex* leaf-cutting ants. *Oecologia* **158**: 165-175 10.1007/s00442-008-1113-z 18668265

[CR2] Bollazzi M. and Roces F. 2002. Thermal preference for fungus culturing and brood location by workers of the thatching grass-cutting ant *Acromyrmex heyeri*. *Insect. Soc.* **49**: 153-157

[CR3] Bolton B. 1994. *Identification Guide to the Ant Genera of the World.* Harvard University Press, Cambridge, MA

[CR4] Brandão C.R.F. and Mayhé-Nunes A.J. 2001. A new fungus-growing ant genus, *Mycetagroicus**gen. nov.* with the description of three new species and comments on the monophyly of the Attini (Hymenoptera: Formicidae). *Sociobiology* **38**: 639-665

[CR5] Brandão C.R.F. and Mayhé-Nunes A.J. 2008. A new species of the fungus-farming ant genus *Mycetagroicus* Brandão and Mayhé-Nunes (Hymenoptera, Formicidae, Attini). *Rev. Bras. Entomol.* ***52***: 349-352

[CR6] Chapela I.H., Rehner S.A., Schultz T.R. and Mueller U.G. 1994. Evolutionary history of the symbiosis between fungus-growing ants and their fungi. *Science* **266**: 1691-1694 10.1126/science.266.5191.1691 17775630

[CR300] Currie C.R., Scott J.A., Summerbell R.C. and Malloch D. 1999. Fungus-growing ants use antibiotic-producing bacteria to control garden parasites. *Nature* **398**: 701-704

[CR7] Diehl-Fleig E. and Diehl E. 2007. Nest architecture and colony size of the fungus-growing ant *Mycetophylax simplex* Emery, 1888 (Formicidae, Attini). *Insect. Soc.* **54**: 242-247

[CR8] Fernández-Marín H., Zimmerman J.K., Wcislo W.T. and Rehner S.A. 2005. Colony foundation, nest architecture and demography of a basal fungus-growing ant, *Mycocepurus smithii* (Hymenoptera, Formicidae). *J. Nat. Hist.* **39**: 1735-1743

[CR9] Gallardo A. 1916. Notes systématiques et éthologiques sur les fourmis attines de la République Argentine. *An. Mus. Nac. Hist. Nat. Buenos Aires* **28**: 317-344

[CR400] Gauld I. and Bolton B. 1988. *The Hymenoptera*. British Museum (Natural History), Oxford University Press, New York

[CR10] Goulet H. and Huber J.T. 1993. *Hymenoptera of the World: an Identification Guide to Families*. Research Branch, Agriculture Canada

[CR11] Green A.M., Mueller U.G. and Adams R.M.M. 2002. Extensive exchange of fungal cultivars between sympatric species of fungus–growing ants. *Mol. Ecol.* **11**:191-195 10.1046/j.1365-294x.2002.01433.x 11856421

[CR12] Hölldobler B. and Wilson E.O. 1990. *The Ants*. The Belknap Press of Harvard University Press, Cambridge, MA

[CR13] Katoh K. and Standley D.M. 2013. MAFFT multiple sequence alignment software version 7: improvements in performance and usability. *Mol. Biol. Evol.* **30**: 772-780 10.1093/molbev/mst010 PMC3603318 23329690

[CR14] Klingenberg C., Brandão C.R.F. and Engels W. 2007. Primitive nest architecture and small monogynous colonies in basal Attini inhabiting sandy beaches of southern Brazil. *Stud. Neotrop. Fauna E.* **42**: 121-126

[CR15] Klingenberg C. and Brandão C.R.F. 2009. Revision of the fungus-growing ant genera *Mycetophylax* Emery and *Paramycetophylax* Kusnezov rev. stat., and description of *Kalathomyrmex n. gen.* (Formicidae: Myrmicinae: Attini). *Zootaxa* **2052**: 1-31

[CR16] Krombein K.V. 1967. *Trap*-*nesting Wasps and Bees: Life Histories, Nests, and Associates*. Smithsonian Press, Washington DC

[CR17] Lanyon W.E. 1986. A phylogeny of the thirty-three genera in the *Empidonax* assemblage of tyrant flycatchers. *Am. Mus. Novit.* **2846**: 1-64

[CR18] Mackay W.P., Maes J.M., Fernandez P.R. and Luna G. 2004. The ants of North and Central America: the genus *Mycocepurus* (Hymenoptera: Formicidae). *J. Insect Sci.* **4**: 7 pp 10.1093/jis/4.1.27 PMC1081568 15861242

[CR19] Mehdiabadi N.J. and Schultz T.R. 2010. Natural history and phylogeny of the fungus-farming ants (Formicidae: Myrmicinae: Attini). *Myrmecol. News* **13**: 37-55

[CR20] Mehdiabadi N.J., Mueller U.G., Brady S.G., Himler A.G. and Schultz T.R. 2012. Symbiont fidelity and the origin of species in fungus-growing ants. *Nature Comm.* **3**: 840 10.1038/ncomms1844 22588302

[CR21] Melo G.A.R. 1996. Notes on the nesting biology of *Melipona capixaba* (Hymenoptera, Apidae). *J. Kansas Entomol. Soc.* **19**: 207-210

[CR22] Mikheyev S., Mueller U. G. and Abbott P. 2006. Cryptic sex and many-to-one co-evolution in the fungus-growing ant symbiosis. *Proc. Natl Acad. Sci. USA* **103**: 10702-10706 10.1073/pnas.0601441103 PMC1502295 16815974

[CR23] Mikheyev A.S., Mueller U.G. and Boomsma J.J. 2007. Population genetic signatures of diffuse coevolution between leaf-cutting ants and their cultivar fungi. *Mol. Ecol.* **16**: 209-216 10.1111/j.1365-294X.2006.03134.x 17181732

[CR24] Mikheyev A.S., Vo T.L. and Mueller U.G. 2008. Phylogeography of post-Pleistocene population expansion in a fungus-gardening ant and its microbial mutualists. *Mol. Ecol.* **20**: 4480-4488 10.1111/j.1365-294X.2008.03940.x 18986494

[CR25] Mikheyev A.S., Mueller U.G. and Abbot P. 2010. Comparative dating of attine ant and lepiotaceous cultivar phylogenies reveals coevolutionary synchrony and discord. *Am. Nat.* **175**: E126-E133 10.1086/652472 20415533

[CR26] Moreira A.A., Forti L.C., Andrade A.P.P., Boaretto M.A.C. and Lopes J.F.S. 2004. Nest architecture of *Atta laevigata* (F. Smith, 1858) (Hymenoptera: Formicidae). *Stud. Neotrop. Fauna E.* **39**: 109-116

[CR27] Moser J.C. 1962. Probing the secrets of the town ant. *Forests & People* **12**: 12-13, 40-41.

[CR28] Moser J.C. and Neff S.E. 1971. *Pholeomyia comans* [Diptera: Milichiidae] an associate of *Atta texana*: Larval anatomy and notes on biology. *Z. Angew. Ent.* **69**: 343-348

[CR29] Moser J.C. 2006. Complete excavation and mapping of a Texas leafcutting ant nest. *Ann. Entomol. Soc. Am.* **99**: 891-897

[CR100] Mueller U.G., Rehner S.A. and Schultz T.R. 1998. The evolution of agriculture in ants. *Science* **281**: 2034-2038 10.1126/science.281.5385.2034 9748164

[CR30] Mueller U.G., Schultz T.R., Currie C.R., Adams R.M.M. and Malloch D. 2001. The origin of the attine ant-fungus mutualism. *Q. Rev. Biol.* **76**: 169-197 10.1086/393867 11409051

[CR31] Mueller U.G. and Gerardo N. 2002. Fungus-farming insects: multiple origins and diverse evolutionary histories. *Proc. Natl Acad. Sci. USA* ***99***: 15247-15249 10.1073/pnas.242594799 PMC137700 12438688

[CR32] Mueller U.G., Ishak H., Lee J.C., Sen R. and Guttel R.R. 2010. Placement of attine ant-associated *Pseudonocardia* in a global *Pseudonocardia* phylogeny (Pseudocardiaceae, Actinomycetales): a test of two symbiont-association models. *Antonie Van Leeuwenhoek* **98**: 195-212 10.1007/s10482-010-9427-3 PMC2975052 20333466

[CR33] Mueller U.G., Mikheyev A.S., Solomon S.E. and Cooper M. 2011a. Frontier mutualism: coevolutionary patterns at the northern range limit of the leaf-cutter ant-fungus symbiosis. *Proc. R. Soc. B* **278**: 3050-3059 10.1098/rspb.2011.0125 PMC3158939 21389026

[CR34] Mueller U.G., Mikheyev A.S., Hong E., Sen R., Warren D.L., Solomon S.E., Ishak H.D., Cooper M., Miller J.L., Shaffer K.A. and Juenger T.E. 2011b. Evolution of cold-tolerant fungal symbionts permits winter fungiculture by leafcutter ants at the northern frontier of a tropical ant-fungus symbiosis. *Proc. Natl Acad. Sci. USA* **108**: 4053-4056 10.1073/pnas.1015806108 PMC3053988 21368106

[CR35] Packer L. 1991. The evolution of social behavior and nest architecture in sweat bees of the subgenus *Evylaeus* (Hymenoptera: Halictidae): a phylogenetic approach. *Behav. Ecol. Sociobiol.* **29**: 153-160

[CR36] Rabeling C., Verhaagh M. and Engels W. 2007. Comparative study of nest architecture and colony structure of the fungus growing ants *Mycocepurus goeldi* and *M. smithii*. *J. Insect Sci.* **7**: 1-13 10.1673/031.007.4001 PMC2999435 20331400

[CR37] Rabeling C. and Bacci M.J. 2010. A new workerless inquiline in the Lower Attini (Hymenoptera: Formicidae), with a discussion of social parasitism in fungus-growing ants. *Syst. Entomol.* **35**: 379-392

[CR38] Ramos-Lacau L.S., Silva P.S.D., Lacau S., Delabie J.H. and Bueno O.C. 2012. Nesting architecture and population structure of the fungus–growing ant *Cyphomyrmex transversus* (Formicidae: Myrmicinae: Attini) in the Brazilian coastal zone of Ilhéus, Bahia. *Annls Soc. Entomol. France* **48**: 439-445

[CR39] Rasmussen C. and Camargo J.M. 2008. A molecular phylogeny and the evolution of nest architecture and behavior in *Trigona* ss (Hymenoptera: Apidae: Meliponini). *Apidologie* **39**: 102-118

[CR40] Sabrosky C.W. 1959. A revision of the genus *Pholeomyia* in North America (Diptera, Milichiidae). *Ann. Entomol. Soc. Am.* **52**: 316-331

[CR41] Schmidt R.S. 1995. The evolution of nest-building behavior in *Apicotermes* (Isoptera). *Evolution* **9**: 157-181

[CR42] Schultz T.R. and Meier R. 1995. A phylogenetic analysis of the fungus-growing ants (Hymenoptera: Formicidae: Attini) based on morphological characters of the larvae. *Syst. Entomol.* **20**: 337-370

[CR43] Schultz T.R., Bekkevold D. and Boomsma J.J. 1998. *Acromyrmex insinuator* new species: An incipient social parasite of fungus-growing ants. *Insect. Soc.* **45**: 457-471

[CR44] Schultz T.R., Solomon S.A., Mueller U.G., Villesen P., Boomsma J.J., Adams R.M. and Norden B. 2002. Cryptic speciation in the fungus-growing ants *Cyphomyrmex longiscapus* Weber and *Cyphomyrmex muelleri* Schultz and Solomon, new species (Formicidae, Attini). *Insect. Soc.* **49**: 331-343

[CR45] Schultz T.R., Mueller U.G., Currie C.R. and Rehner S.A. 2005. Reciprocal illumination: A comparison of agriculture in humans and fungus-growing ants. In: *Insect*-*Fungal Association: Ecology and Evolution* (Vega F. and Blackwell M., Eds), New York: Oxford University Press. pp 149-190

[CR46] Schultz T.R. and Brady S.G. 2008. Major evolutionary transitions in ant agriculture. *Proc. Natl Acad. Sci. USA* **105**: 5435-5440 10.1073/pnas.0711024105 PMC2291119 18362345

[CR200] Sen R., Ishak H.D., Kniffin T.R. and Mueller U.G. 2010. Construction of chimaeric gardens through fungal intercropping: a symbiont choice experiment in the leafcutter ant *Atta texana* (Attini, Formicidae). *Behav. Ecol. Sociobiol.* **64**: 1125-1133

[CR47] Silva L.A.G.C. 2007. Biomas Presentes no Estado do Tocantins. In *Consultoria Legilativa Nota Técnica* Câmara dos Deputados, Brasilia, DF, Brasil. 2-9

[CR48] Snodgrass R.E. 1910. The thorax of the Hymenoptera*. Proc. US Nat. Mus.* **39**: 37-91

[CR49] Solomon S.E., Mueller U.G., Schultz T.R., Currie C.R., Price S.L., Oliveira da Silva-Pinhati A.C., Bacci M. and Vasconcelos H.L. 2004. Nesting biology of the fungus growing ants Mycetarotes Emery (Attini, Formicidae). *Insect. Soc.* **51**: 333-338

[CR50] Solomon S.E., Lopes C.T., Mueller U.G., Rodrigues A., Sosa-Calvo J., Schultz T.R. and Vasconcelos H.L. 2011. Nesting biology and fungiculture of the fungus–growing ant, *Mycetagroicus cerradensis*: New light on the origin of higher attine agriculture. *J. Insect Sci.* **11**: 1-12 10.1673/031.011.0112 PMC3281386 21526926

[CR51] Sosa-Calvo J. and Schultz T.R. 2010. Three remarkable new fungus-growing ant species of the genus *Myrmicocrypta* (Hymenoptera: Formicidae), with a reassessment of the characters that define the genus and its position within the Attini. *Ann. Entomol. Soc. Amer.* **103**: 181-195

[CR52] Swann J.E. 2010. Milichiidae (Milichiid flies). 1125-1136. In: *Manual of Central American Diptera*. Vol 2 (Brown B.V., Borkent A., Cumming J.M., Wood D.M., Woodley N.E., Zumbado M.A., Eds), National Research Council of Canada. 1442 pp

[CR53] Tschinkel W.R. 2003. Subterranean ant nests: trace fossils past and future? *Palaeogeogr. Palaeocl.* **192**: 321-333

[CR54] Tschinkel W.R. 2011. Back to basics: sociometry and sociogenesis of ant societies (Hymenoptera: Formicidae). *Myrmecol. News* **14**: 49-54

[CR55] Tulloch G.S. 1935. Morphological studies of the thorax of the ant. *Entomol. Am*. **15**: 93-131

[CR56] Vo T.L., Mikheyev A.S. and Mueller U.G. 2009. Free-living fungal symbionts (Lepiotaceae) of fungus-growing ants (Attini: Formicidae). *Mycol.* **101**: 206-210 10.3852/07-055 19397193

[CR57] Waller D.A. 1980. Leaf-cutting ants and leaf-riding flies. *Ecol. Entomol.* **5**: 305-306

[CR58] Weber N.A. 1972. Gardening ants, the attines. *Philadelphia: The American Philosophical Society*. 146 pp

[CR59] Wenzel J.W. 1998. A generic key to the nests of hornets, yellow jackets, and paper wasps worldwide (Vespidae: Vespinae, Polistinae). *Am. Mus. Novit.* **3224**: 1-39

[CR60] Wheeler G.C. and Wheeler J. 1976. Ant larvae: Review and synthesis. *Mem. Entomol. Soc. Washington* **7**: 1-108

[CR61] Wild A.L. and Brake I. 2009. Field observations on *Milichia patrizii* ant-mugging flies (Diptera: Milichiidae: Milichiinae) in KwaZulu-Natal, South Africa. *Afr. Invertebr.* **50B:** 205-212

[CR62] Zyskowski K. and Prum R.O. 1999. Phylogenetic analysis of the nest architecture of Neotropical ovenbirds (Furnariidae). *The Auk* **116**: 891-911

